# Innovative problem-solving in wild hyenas is reliable across time and contexts

**DOI:** 10.1038/s41598-020-69953-5

**Published:** 2020-08-03

**Authors:** Lily Johnson-Ulrich, Kay E. Holekamp, David Z. Hambrick

**Affiliations:** 1 Department of Integrative Biology, Michigan State University, 288 Farm Lane, Rm 203, Natural Sciences Bldg, East Lansing, MI 48824 USA; 2 Ecology, Evolutionary Biology, and Behavior Program, Michigan State University, East Lansing, MI 48824 USA; 3 Department of Psychology, Michigan State University, East Lansing, MI 48824 USA

**Keywords:** Behavioural ecology, Animal behaviour

## Abstract

Individual differences in behavior are the raw material upon which natural selection acts, but despite increasing recognition of the value of considering individual differences in the behavior of wild animals to test evolutionary hypotheses, this approach has only recently become popular for testing cognitive abilities. In order for the intraspecific approach with wild animals to be useful for testing evolutionary hypotheses about cognition, researchers must provide evidence that measures of cognitive ability obtained from wild subjects reflect stable, general traits. Here, we used a multi-access box paradigm to investigate the intra-individual reliability of innovative problem-solving ability across time and contexts in wild spotted hyenas (*Crocuta crocuta*). We also asked whether estimates of reliability were affected by factors such as age-sex class, the length of the interval between tests, or the number of times subjects were tested. We found significant contextual and temporal reliability for problem-solving. However, problem-solving was not reliable for adult subjects, when trials were separated by more than 17 days, or when fewer than seven trials were conducted per subject. In general, the estimates of reliability for problem-solving were comparable to estimates from the literature for other animal behaviors, which suggests that problem-solving is a stable, general trait in wild spotted hyenas.

## Introduction

The questions of how and why cognition evolves across the animal kingdom remain unresolved despite more than a century of intensive research. The most common approach to addressing these questions has been to compare average levels of cognitive performance among species^1–5^. In this *interspecific* approach, individual differences within species are treated as random error (or “noise”). Recently, there has been growing recognition of the value of using individual differences to test evolutionary hypotheses—the *intraspecific* approach^6^. Intraspecific studies of free-ranging populations are especially valuable for understanding cognitive evolution, because individual variation is the raw material on which natural selection acts. This approach allows researchers to examine the causes of cognitive variation in an ecologically valid context and also to examine the fitness consequences of this variation^7–9^. Despite this recognition, in the field of cognitive ecology there have been few attempts to empirically test the hypothesis that measures of cognition reflect stable, general traits, meaning traits expected to influence performance across time and across a wide range of situations^10,11^.

The hypothesis that a cognitive measure reflects a stable, general trait predicts that the measure should have a high degree of reliability: an animal that receives a high score on the measure at one point in time and in one context should receive a high score at later points in times and in other contexts, and the performance of animals receiving low scores on the measure should be similarly consistent*.* As a psychometric concept, *reliability* refers to the amount of error contained in a measure, as reflected in the stability of the measure across contexts and time. Although reliability is synonymous with the term ‘repeatability’, which refers to consistent individual differences in the behavior of non-human animals^12^, we use reliability because it is a well-defined psychometric term used in diverse literatures on individual differences in psychological traits, including cognitive abilities in both humans and non-human animals. It is especially important to demonstrate reliability of measures reflecting animal cognition in the wild, because there are many potential sources of error, including both external factors (e.g., weather, presence of conspecifics) and internal factors (e.g., hunger, stress)^9,13–15^.

In this study, we assessed the intra-individual reliability of *innovativeness* across time and context in wild spotted hyenas using a problem-solving paradigm. Defined as the ability to solve a novel problem or use a novel behavior to solve a familiar problem, innovativeness is among the most commonly measured cognitive abilities in non-human animals^16^. Although there has been a great deal of interest in the relationship between innovativeness and variables such as brain size, ability to invade new habitat, and life history traits in a diverse array of taxa, formal attempts to evaluate the reliability of problem-solving paradigms used to measure innovativeness remain very rare. In a meta-analysis, Cauchoix et al.^11^ identified only six publications reporting reliability for any measure of cognitive performance,of these only two measured innovative problem-solving in wild subjects, and both were in birds. Thus, there is a pressing need to examine the reliability of innovative problem-solving in other wild animals, especially in wild mammals. Furthermore, most studies only measure cognition at two time points and across two to four different tasks^17–20^, and there has been very little research examining how reliability might vary based on the number of measures or the length of the interval between measures, nor how reliability might vary within species among different age-sex classes (e.g. Ref.^13^. Our ignorance here is due, in part, to the numerous logistical challenges of experimentally measuring innovativeness repeated times in the same subjects—a problem that is particularly pronounced in wild subjects where locating and enticing individuals to perform cognitive tests even once can be difficult, and tracking individuals for repeated testing may be impossible in many species. However, for the intraspecific approach in wild animals to be useful for testing evolutionary hypotheses, researchers *must* provide evidence that measures of innovative problem-solving reflect stable traits, and that estimates of reliability are robust against numerous sources of variation in testing environment and methodology.

The spotted hyena is well-established as a model organism for testing hypotheses about the evolution of cognition^21^ and innovativeness has previously been measured in both captive and wild hyenas^22–24^, but the problem-solving paradigms used to measure innovativeness were never previously tested for reliability except by Johnson-Ulrich et al.^25^. Here we measured reliability (R) by calculating intraclass correlation coefficients (ICC), which are commonly used in behavioral ecology to assess the reliability of behavioral traits within individuals^26^. An ICC estimates the amount of variation in the response variable explained by random effects or grouping factors in mixed hierarchical models. Ultimately, we found significant reliability for problem-solving performance in wild spotted hyenas and demonstrate how estimates of reliability vary across tasks, trials, age-sex classes, the temporal interval between observations, and the total number of observations.

## Results

**Figure 1:**
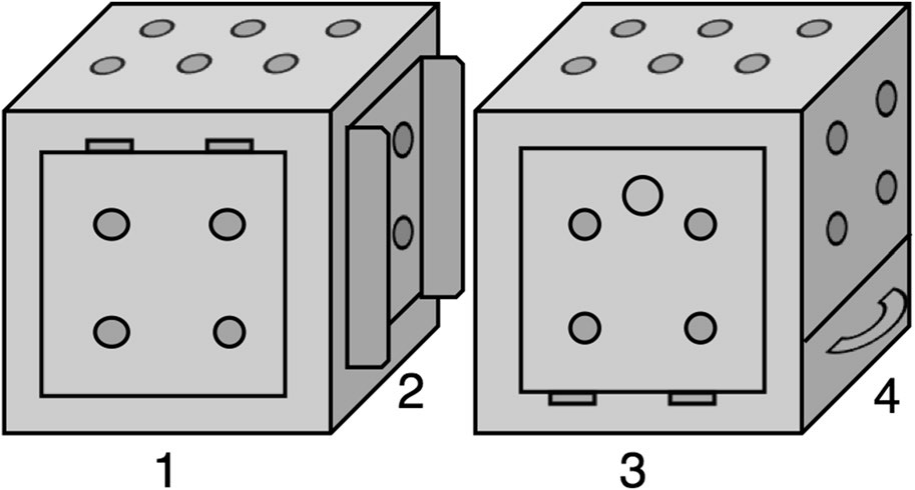
The MAB used in the current study. (1) The push flap solution; (2) the sliding door solution; (3) the pull flap solution; and (4) the drawer solution. Small filled grey circles indicate the approximate number and location of holes drilled through the wall of the MAB. Large gray circle on side 3 represents the location of the doorknob. Small rectangles represent the location of door hinges. Reproduced with permission from Johnson-Ulrich et al.^23^.

**Figure 2:**
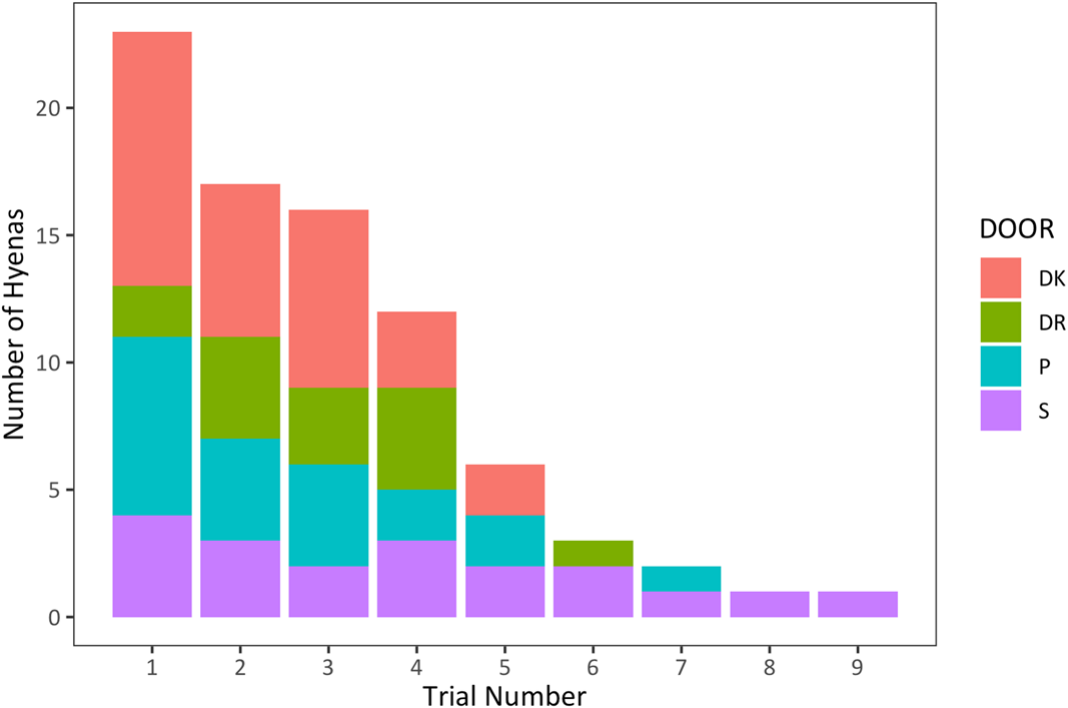
The number of hyenas using each solution across their successful trials within Phase 1 of testing. Twenty-three hyenas were successful on at least one trial, but within these twenty-three hyenas the number of successful trials they had within Phase 1 varied. *DK* door knob, *DR* drawer, *P* push door, *S* sliding door.

Seventy-two hyenas participated in 694 test trials with a multi-access box (MAB). MABs are problem-solving paradigms used to measure innovativeness. The MAB used in the present study was a metal box with one of four different doors on each vertical face (Fig. 1). Each door required a unique motor behavior to open, but all four doors opened to the same common interior from which a hyena could retrieve bait. Hyenas were given repeated trials, and after a hyena opened the same door on three of four consecutive test trials, that door was blocked, forcing the hyena to open a different door to retrieve the bait (Fig. 2). Testing was thus divided into four ‘phases’ in which hyenas were required to use four different doors to open the MAB (Supplementary Fig. S1). Overall, our sample included an adequate representation of each age and sex class with 17 adult females, seven adult males, 13 subadult females, 17 subadult males, 10 female cubs, and nine male cubs. Out of these 72 hyenas, 23 opened the MAB at least once (mean = 2.74 doors, SD = 1.39) and 11 opened each of the four doors to the MAB at least once across their trials. Because we collected data with more hyenas that never opened the MAB (N = 49 hyenas, n = 376 trials) than data with hyenas who opened the MAB at least once (N = 23 hyenas, n = 318 trials), and because including the former hyenas would lead to zero-inflation and an inflated reliability estimate, we excluded data from hyenas that never opened the MAB from our analyses. Instead, we only assessed the reliability of problem-solving for hyenas that opened the MAB at least once. Among these 23 hyenas our sample included five adult females, two adult males, five subadult females, six subadult males, one female cub, and four male cubs.

### Contextual reliability of problem-solving ability across different doors

Contextual reliability is typically assessed by comparing performance across different tasks that measure the same cognitive ability^11,27^. Because each of the four doors to the MAB required a different motor behavior, we first investigated the contextual reliability of problem-solving performance with a model examining the likelihood of solving each of the four different MAB doors (door knob, slot, push, drawer) at least once. Because innovation is defined as using a novel behavior to solve a problem^16^, hyenas only ‘innovated’ when they solved each door of the MAB for the very first time. Thus, our estimate of reliability for problem-solving across doors provides a valuable indication of the reliability of ‘innovativeness’, in the strictest sense, among hyenas. In this model our response variable was a binary variable indicating whether or not a hyena had solved each of the four doors to the MAB at least once (Table 1: Model 1). Each hyena received four dichotomous scores for each of the four unique MAB doors, with a score of one indicating that they solved a door at least once and a score of zero indicating that they never solved that particular door despite contacting the MAB on multiple trials. We included age class as a fixed effect in this model (see “Statistical analysis”); hyenas in the cub (GLMM: Odds ratio = 0.05, P = 0.058) and subadult (GLMM: Odds ratio = 0.07; P = 0.055) age classes were less likely than adults to solve each door. Reliability was determined by calculating adjusted ICCs with the R package rptR^28^. Adjusted ICC values are calculated as a ratio of the random effects variance to the combined random effects and residual variance. Of the 23 hyenas that opened the MAB at least once, we found that problem-solving performance across doors was moderately but significantly reliable (Likelihood ratio test: R = 0.40, P = 0.001; Table 1: Model 1). Thus, problem-solving ability was significantly reliable when assessed in the variable contexts of the MAB’s four doors, each of which required a unique solution.

**Table 1:** Reliability results for innovative problem-solving. ‘R’ is the adjusted reliability estimate for subject ID.

	Model description	R	SE	P	N	n	Var_F_	Var_R_
Model 1	Across doors	0.40	0.17	0.001*	23	92	0.17	0.33
Model 2	Across trials	0.18	0.07	< 0.001*	23	318	0.08	0.16
Model 3.1	Females	0.21	0.11	0.005*	11	153	0.20	0.16
Model 3.2	Males	0.14	0.08	0.009*	12	165	0.14	0.12
Model 3.3	Adults	0.07	0.07	0.108	7	125	0.10	0.06
Model 3.4	Subadults	0.24	0.13	0.001*	11	131	0.03	0.24
Model 3.5	Cubs	0.33	0.21	0.005*	5	62	0.02	0.32
Model 4.1	< 1 day	0.93	0.01	< 0.001*	20	157	0.01	0.92
Model 4.2	1–3 days	0.43	0.21	0.008*	18	53	0.16	0.36
Model 4.3	4–16 days	0.33	0.19	0.039*	18	41	0.08	0.30
Model 4.4	> 17 days	0.10	0.11	0.235	20	44	0.06	0.09
Model 5.1	First 2 Trials	0.08	0.20	0.330	23	46	0.09	0.08
Model 5.2	First 3 Trials	0.21	0.16	0.070	22	66	0.05	0.20
Model 5.3	First 4 Trials	NA	NA	NA	22	88	NA	NA
Model 5.4	First 5 Trials	NA	NA	NA	21	105	NA	NA
Model 5.5	First 6 Trials	0.07	0.06	0.138	21	126	0.01	0.07
Model 5.6	First 7 Trials	0.13	0.08	0.0261*	18	126	0.10	0.12
Model 5.7	First 8 Trials	0.20	0.10	0.002*	17	136	0.13	0.17
Model 5.8	First 9 Trials	0.17	0.09	0.003*	16	144	0.11	0.15
Model 5.9	First 10 Trials	0.17	0.10	0.004*	14	139	0.21	0.13

Adjusted R values are calculated as a ratio of the random effects variance over the random effects and residual variance. * are used to indicate P values that are significant at α < 0.05. ‘N’ is the number of subjects included in each model and ‘n’ is the total number of observations included in the model. ‘Var_F_’ is the relative amount of variation explained by fixed effects compared to total variation. ‘Var_R_’ is the relative amount of variation explained by random effects compared to total variation. Models 5.3 and 5.4 have NAs because the variance explained by the random effect subject ID was too small to be estimated in these models.

### Temporal reliability of problem-solving across trials

In addition to contextual reliability, the temporal reliability of cognitive traits is commonly assessed by comparing performance across repeated trials with the same cognitive test (e.g. problem-solving: Refs.^11,17,19,28^. Because problem-solving performance was moderately reliable across different doors, we next examined the reliability of problem-solving performance across each subject’s trials, regardless of the specific doors used, in order to investigate temporal reliability. We gave each hyena multiple trials with the MAB in order to give subjects the opportunity to solve its different doors and examine performance across different phases of testing. Although hyenas are not strictly ‘innovating’ when they open a MAB door that they’ve previously solved, most studies on innovative problem-solving conduct repeated trials to compare the acquisition of innovations across individuals or assess their spread through populations (e.g. Refs.^21,28–32^, so investigating the reliability of problem-solving performance across trials is relevant for future research.

In this model, and all subsequent models, our response variable was a binary variable indicating whether a hyena opened or failed to open a door of the MAB, irrespective of which specific door it was working on. On average hyenas were successful in 54.5% of trials (SD = 27.0%, N = 23 hyenas, n = 318 trials). Because temporal reliability may be influenced by learning and experience^11^ we included a fixed effect of trial number in order to control for the number of previous trials in which each hyena participated. We also included age class and phase number as fixed effects (see “Statistical analysis”). We found that cubs (GLMM: Odds ratio = 0.31, P = 0.096) and subadults (GLMM: Odds ratio = 0.32, P = 0.046) were both less likely than adults to have successful trials with the MAB. Hyenas were also less likely to solve the MAB at later than earlier phases of testing (GLMM: Odds ratio = 0.54, P = 0.044), which probably represents the increasing difficulty across phases. Trial number had a significant positive effect on the odds of a trial being successful (GLMM: Odds ratio = 1.11, P = 0.050), which suggests that prior experience or learning with the MAB was important, but the effect of trial number on the odds of success was relatively small compared to the effects of age class and phase. Furthermore, these fixed effects only explained half as much variation in the response variable (Var_F_ = 0.08; Table 1: Model 2) as that explained by hyena ID (Var_R_ = 0.16; Table 1: Model 2). Among the 23 hyenas that opened the MAB at least once, problem-solving performance was significantly reliable (Likelihood ratio test: R = 0.18, P < 0.001; Table 1: Model 2). This result suggests that hyenas’ problem-solving performance was generally consistent across trials, even after controlling for the number of previous trials, the phase of testing, and the hyena’s age class.

### Reliability of innovative problem-solving within different age-sex classes

**Figure 3:**
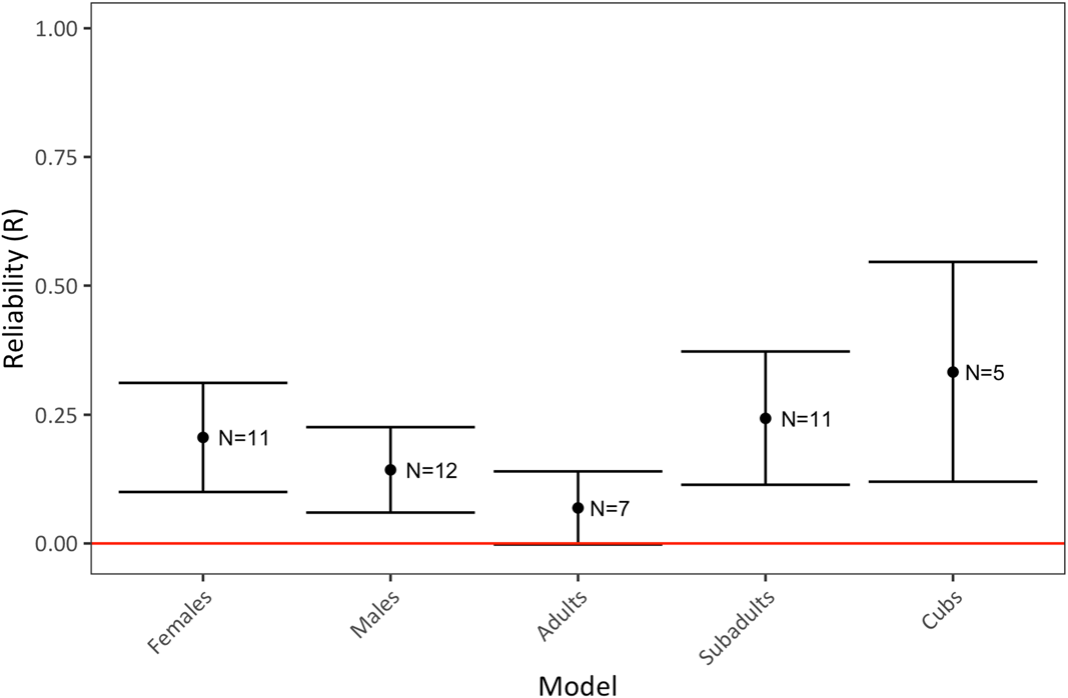
Reliability of problem-solving success within different age and sex classes. R values are calculated as adjusted repeatability ratios where the variance explained by fixed effects is not included in the denominator. Error bars show standard error. Standard error was estimated using parametric bootstrapping (N = 1,000) from the R package rptR. N indicates the number of subjects included in each model.

Next, we inquired whether temporal reliability varied among individuals in different age-sex classes. For example, some evidence suggests that female animals exhibit more reliable behavior than males^12^. Furthermore, it seems reasonable to expect that juveniles, which are still developing, might exhibit behavior that is less reliable than that of adults in addition to showing slightly worse performance with the MAB than adults. To compare reliability within different age and sex classes we partitioned our dataset into five different categories: females, males, adults, subadults, and cubs in order to create five different models examining reliability for each age-sex class independently. We included age class as a fixed effect in the female-only and male-only models, and we also included both trial number and phase of testing as fixed effects in all models (see “Statistical analysis”). We did not include sex as a fixed effect in each age class model because problem-solving did not vary with sex (Supplementary Tables S2, S3). We found that most age and sex classes showed moderate levels of reliability (Likelihood ratio test: R = 0.21–0.33, P < 0.001; Table 1: Models 3.1–3.5; Fig. 3) with the exception of adult hyenas, for which the reliability of problem-solving was not significantly different than zero (Likelihood ratio test: R = 0.07, P = 0.11; Table 1: Model 3.3).

### Reliability of innovative problem-solving across different timespans

**Figure 4:**
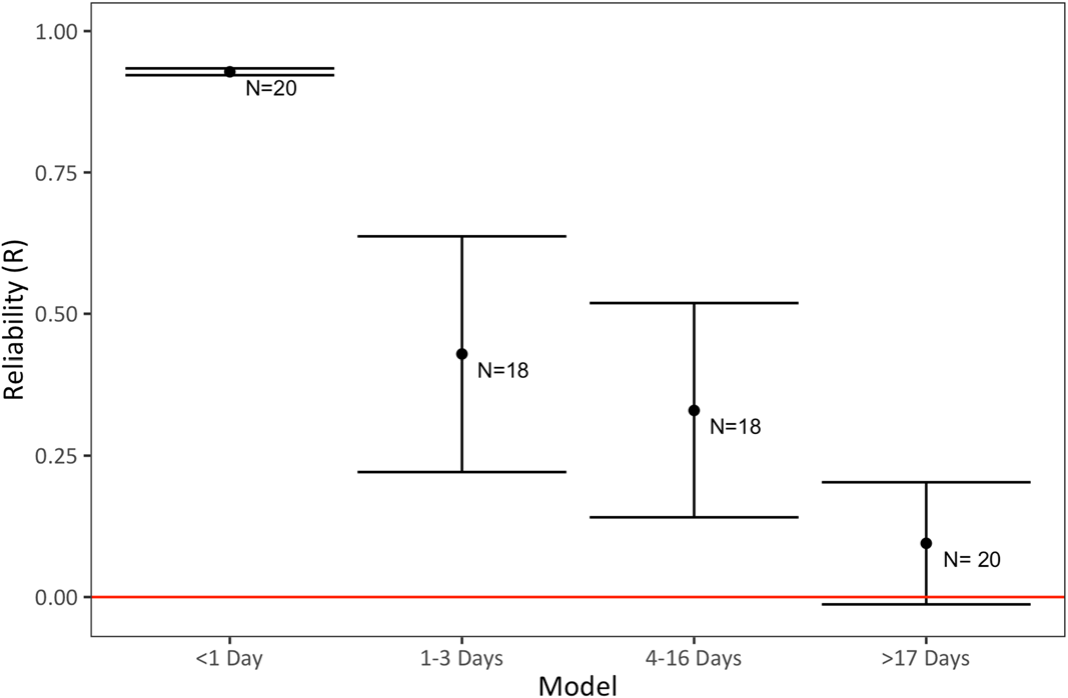
Reliability of problem-solving success across different timespans. R values are calculated as adjusted repeatability ratios where the variance explained by fixed effects is not included in the denominator. Error bars show standard error. Standard error was estimated using parametric bootstrapping (N = 1,000) from the R package rptR. N indicates the number of subjects included in each model.

Although most test trials within subjects (49.37%) were conducted less than 1 day apart, the average number of days between trials was 19.41 ± 56.00 days (median = 0, range = 0–301 days). We were interested in whether temporal reliability between any given trial and the trial that followed it was affected by the amount of time between trials. To do this, we created a dataset where we paired each subject’s trial with the trial that followed it and calculated the number of days elapsing between the two trials. We next partitioned this dataset into trials that occurred less than one day apart, one to three days apart, four to sixteen days apart, and more than 17 days apart. The number of bins and the date range included in each bin were chosen to distribute the number of trials across each date range as equally as possible. We then calculated reliability between pairs of trials for each of these datasets (Table 1: Models 4.1–4.4; Fig. 4). We included age class, phase of testing, and trial number in these models (see “Statistical analysis”). We found that reliability was extremely high for trials collected on the same day (Likelihood ratio test: R = 0.93, P < 0.001; Table 1: Model 4.1), but reliability became non-significant when trials were separated by 17 or more days (Likelihood ratio test: R = 0.10, P = 0.235; Table 1: Model 4.4).

### Reliability of innovative problem-solving across different numbers of trials

**Figure 5:**
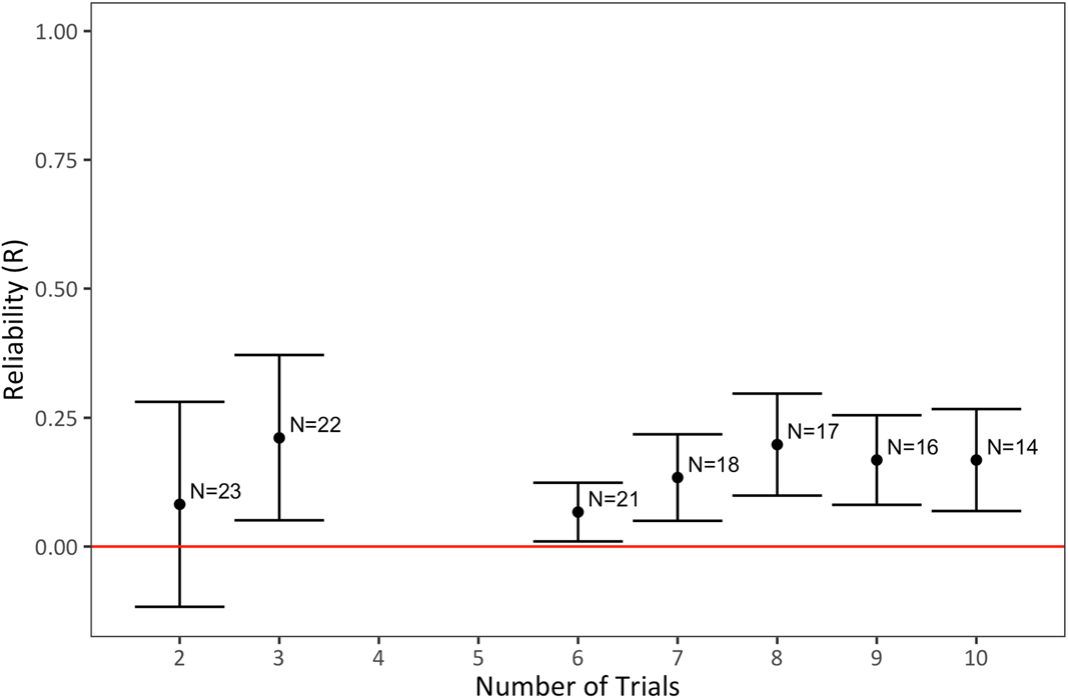
Reliability of problem-solving success across different numbers of trials. R values are calculated as adjusted repeatability ratios where the variance explained by fixed effects is not included in the denominator. Error bars show standard error. Standard error was estimated using parametric bootstrapping (N = 1,000) from the R package rptR. N indicates the number of subjects included in each model.

Finally, we were interested in how the varying number of trials collected per hyena might affect estimates of temporal reliability. On average, hyenas received 13.8 ± 7.3 trials (median = 15 trials, range = 2–26 trials). Although we found modest levels of temporal reliability when we included every trial in Model 2 (Table 1), we were interested in how our estimates might have changed if we’d only sampled hyenas a set number of times. Collecting a larger number of trials per hyena could, in theory, increase the accuracy of estimates about their problem-solving ability and therefore increase reliability; however, increasing the number of trials can also strengthen learning and memory, which may ultimately reduce estimates of reliability if all individuals eventually converge at a high level of performance^11^. On the other hand, because we were testing hyenas in the wild, larger number of trials were also more likely to take place across different testing sessions, different timespans, and under variable environmental conditions which could, in theory, decrease estimates of reliability due to increased variability with increasing numbers of trials. To estimate reliability for varying numbers of trials, we calculated reliability for hyenas in nine models where we included only their first two to ten trials. We found that estimates for the reliability of problem-solving performance were not significantly greater than zero until we had sampled each hyena seven times (Likelihood ratio test: R = 0.13, P = 0.026, Table 1: Model 5.6; Fig. 5). With seven or more trials estimates of reliability were modest, but nonetheless significantly greater than zero (R = 0.13–0.20; Table 1: Models 5.6–5.9; Fig. 5).

## Discussion

Overall, our results suggest that innovative problem-solving ability is a stable, general trait in wild spotted hyenas. Our estimates for the reliability of problem-solving performance are comparable to the average reliability of other behaviors in wild animals^12^, and also to the average reliability of other cognitive measures in both captive and wild animals^11^. However, building on previous findings, we further present evidence that, with a few important exceptions, problem-solving performance is reliable across context, time, age-sex class, the interval between observations, and the number of observations.

We found moderate levels of reliability for problem-solving performance across the four different MAB doors. These doors represent four different motor tasks, each designed to measure innovativeness, and we found that hyenas who innovated with one door to the MAB were moderately likely to innovate with the other three doors to the MAB (Table 1: Model 1). This result is similar to studies in wild and captive birds that have generally found consistent performance among problem-solving tasks requiring different motor actions^18,19,35,36^.

**Figure 6:**
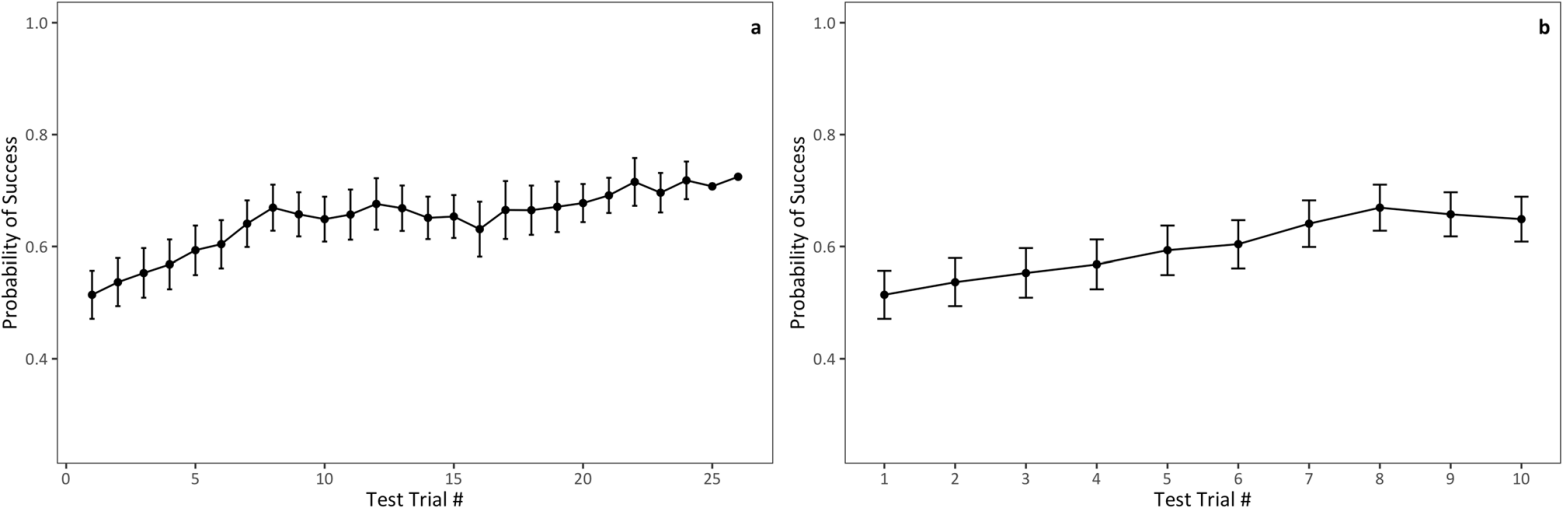
The predicted probabilities of a successful trial with the MAB as a product of trial number from a binomial GLMM (Table 1: Model 2). Error bars indicate standard error. (**a**) Shows the log odds of successfully opening the MAB as a function of test trial number. (**b**) Shows the predicted probabilities of a successful trial with the MAB as a product of trial number for hyenas with at least 10 trials.

Next, we also evaluated the temporal reliability of problem-solving performance across all trials, irrespective of the specific door used to open the MAB. We found a modest, but significant, level of reliability for problem-solving performance across trials (Table 1: Model 2). Because trials were conducted across a wide variety of socio-ecological conditions we were impressed to find hyenas demonstrate even this level of consistency in performance. Trial number did have a significant effect in this model, which suggests that learning may have played a role in shaping consistency across trials (Fig. 6); however, the amount of variation explained by subject ID in these models was twice that explained by the fixed effects, which included trial number. This result is also consistent with other cognitive studies; a meta-review of the reliability of cognitive abilities similarly found that repetition number usually had an important effect on cognitive performance^11^. However, this meta-review also found that adjusting estimates of R for repetition number usually did not increase R, so the authors concluded that repetition numbers largely had negligible effects on estimates of temporal reliability. Likewise, in most of our models our adjusted R values were only modestly larger than the total amount of variation explained by the random effects. While most studies of problem-solving performance do provide evidence that subjects improve their performance over trials, this improvement is typically gradual, which suggests that subjects do not perfectly remember the motor behaviors used to innovate during their first trial^14,22,24,30,34^. Instead, the literature suggests that behaviors such as motor diversity or flexibility may be key for successful problem-solving and that these behaviors, even though they might interact with memory, are independent from learning^34,37–39^. Ultimately, a great deal of variation in problem-solving performance was left unexplained by our models, an unsurprising result given that our subjects were wild, free-ranging hyenas tested in an uncontrolled environment. Future research investigating this remaining variation may shed light on the various individual behaviors or socio-ecological conditions that favor successful problem-solving.

Next, we examined the reliability of problem-solving performance within different age-sex classes. Both female and male hyenas showed similar levels of reliability for problem-solving performance (Table 1: Models 3.2–3.3) with a slight trend towards higher reliability in females. These results are similar to results for behavior across animals more generally; a meta-review of the reliability of animal behavior found that females tend to show slightly more reliable behavior than males when mate-preference behavior is excluded^12^. When we compared the reliability of problem-solving performance across hyena age classes, we found significant reliability for problem-solving performance in subadults and cubs, but problem-solving performance was not significantly reliable for adults. This result is the opposite of what we’d expected, especially because subadults and cubs were significantly less likely to solve the MAB. A meta-review of the reliability of animal behavior found that adults and juveniles tend to show similar levels of reliability across behaviors^12^. In wild hyenas, it may be that adults must contend with a wider variety of distractions than non-reproductively active individuals that are still largely reliant on maternal support for survival^40^. However, it may also be that higher reliability among cubs and subadults compared to adults is directly related to their poorer performance with the MAB compared to adults. Cubs and subadults were successful on 45.8 ± 32.3% and 47.4 ± 27.6% of trials respectively whereas adults were successful on 72.1% ± 13.7% of trials. In adults, lower reliability here could be a result of a ceiling effect where the relatively high success rate and lower variability across trials in adults reduces the amount of variation explained by individual differences.

In general, estimates of reliability are higher for behavioral observations that are made closer together in time^12^. Here, we found remarkably high reliability for problem-solving performance within pairs of trials separated by less than a day (Table 1: Model 4.1). We also found low to moderate reliability for trials separated by as much as 16 days (Table 1: Model 4.2–4.3). Only when trials were separated by more than 17 days did we find no significant reliability within pairs of trials (Table 1: Model 4.4). The lack of reliability among pairs of trials separated by 17 days or more could reflect a limitation of hyenas’ long-term memory, but research with wild spotted hyenas suggests that they are able to efficiently open a previously solved puzzle box even after delays of over a year (unpublished data). In addition to memory, both internal and external environmental conditions (e.g. hunger, social environment) are also much more likely to vary across larger than shorter time spans. That hyenas still show some consistency even with as much as two weeks separating trials is important because it can be extremely difficult to consistently locate subjects for repeated testing, especially in animals like spotted hyenas that live in fission–fusion societies occupying large territories.

In a meta-review of reliability in earlier animal behavior research, reliability estimates were generally not affected by the number of observations per individual^12^. Here, we found low to no reliability for problem-solving performance when fewer than seven trials were conducted per individual. Part of this result may be due to sample size, with just 23 hyenas that solved the MAB at least once, we were only able to include 46 trials in Model 5.1 (Table 1). However, part of this may also reflect high intra-individual variability in problem-solving performance for subjects in their first several trials. Although most hyenas opened the MAB on their first trial (median = 1 trial, mean ± SD = 1.96 ± 1.26 trials), the highest trial number in which any of these subjects opened the MAB for the very first time was the fourth trial. No subjects ever solved the MAB after four consecutive failures, despite having subsequent opportunities to do so. For this reason we used a conservative criterion of at least five consecutive failures to classify hyenas as non-innovative (N = 49 hyenas, n = 376 trials), though their trials were not included in our models examining reliability. The lack of reliability across our subjects’ early trials differs from the results obtained from a meta-review of reliability of animal behavior generally, and probably reflects the difficulty of getting accurate measures of animal cognition, especially in wild subjects, where many other internal and external factors may affect the way a subject interacts with a test apparatus, independent of its actual cognitive abilities. Our results suggest that, if researchers are testing problem-solving in wild subjects, they should aim to collect many trials per subject to ensure accurate estimates of their problem-solving ability, and aim to identify a minimum number of trials per subject for inclusion in analyses. In hyenas, it appears that 5–7 trials per subject may be required to observe consistent individual differences in their problem-solving ability. In total, we deployed the MAB an average of 88.5 ± 34.72 (N = 4 clans) times in each of four study groups in order to identify initial successful trials for all 23 innovative hyenas (see “Test protocol”).

Our study offers an important demonstration of the reliability of innovative problem-solving in a wild mammal. However, reliability does not necessarily correlate with validity. Previous research has debated the conceptual validity of problem-solving paradigms for measuring innovativeness^14,37,41,42^. Although this debate is not entirely settled, researchers have found that the behaviors leading to spontaneous innovations in the wild are very similar to the behaviors that underlie experimentally-observed innovations using problem-solving paradigms^37^, which strongly suggests that problem-solving paradigms are valid for measuring innovativeness. However, it is also important to consider the ecological validity of a paradigm and tasks should be designed with a species’ underlying capabilities in mind. We designed a multi-access box that required spotted hyenas to use behaviors that are part of their natural foraging repertoire to solve a novel problem for a food reward. This kind of puzzle box is sometimes called a novel extractive foraging puzzle because it requires subjects to extract food from a container. Spotted hyenas are dietary generalists and mammalian bones, which represent an important part of their diets, require a moderate degree of extractive foraging to access the marrow within. Therefore, it is not surprising that spotted hyenas were able to innovate with this kind of problem-solving paradigm. However, for animals that never use extractive foraging in the wild, problem-solving paradigms like the one used in the current study might not be ecologically valid tools for assessing innovativeness.

In conclusion, it appears that, even with the many challenges posed by testing animals in the wild, we were nevertheless able to reliably measure innovative problem-solving ability in hyenas. Overall, our results on reliability complement the literature on the validity of innovative problem-solving paradigms, and we conclude that innovative problem-solving paradigms are reliable tools for measuring individual variation in cognitive performance.

## Methods

### Study site and subjects

We tested innovativeness in four neighboring spotted hyena clans within the Maasai Mara National Reserve, Kenya between June 2016 and November 2017. These clans ranged in size from 30 to 55 adult hyenas. Spotted hyena clans represent distinct social groups that are made up of multiple unrelated females, their offspring, and adult immigrant males. Clans are structured by strict linear dominance hierarchies, with an alpha female and her offspring at the top, followed by lower-ranking females and their offspring, with adult immigrant males occupying the lowest rank positions. Births occur year-round and unrelated females raise their offspring together at a communal den. Female hyenas stay in their natal clan throughout their lives, whereas male hyena usually disperse to join new clans when they are 24–60 months old, after they reach sexual maturity^43,44^.

All subjects were identified by their unique spot patterns and ear damage. Hyenas of all age classes and both sexes were included in the study. All subjects were sexed within the first few months of life based on genital morphology^40^. Age classes were based on life history stage^45^. Cubs were defined as hyenas that were still dependent on the communal den for shelter; on average, Mara cubs become den-independent around 9–12 months of age^45^. Subadults were hyenas who were den-independent but had not yet reached sexual maturity. Adults were hyenas that had reached sexual maturity. In females, sexual maturity was determined by the observation of mating, visual evidence of first parturition, or the female reaching three years of age, whichever came first^46^. In males, sexual maturity was determined by dispersal status, males who were still present in their natal clan at testing were classified as subadults and immigrant males were classified as adults.

### Multi-access box paradigm for measuring repeated innovation

We tested innovativeness in wild spotted hyenas using a multi-access box designed for use with mammalian carnivores^24^. The multi-access box (hereafter, ‘the MAB’) is a problem-solving paradigm, also known as an artificial or novel extractive foraging task, where subjects must solve a novel problem to obtain a food reward. In contrast to traditional problem-solving tasks, MAB paradigms typically offer multiple solutions to the same puzzle, each requiring its own unique behavior pattern. As a condensed battery of tasks, the MAB paradigm allows researchers to measure innovation, not just once, but multiple times across different solutions^47^. We chose to use a MAB paradigm because it allowed us to compare reliability across repeated trials within the same solution to reliability across different solutions. Reliable success with the same solution across trials may be a result of individual learning rather than a result of a stable cognitive trait. However, if individuals reliably innovate by opening multiple unique solutions to the MAB this would suggest that innovativeness is a stable cognitive trait. The MAB in the current study was a steel box, measuring 40.64 × 40.64 × 40.64 cm (length × width × height), with four unique doors, each requiring a different motor behavior, that could be used to access a common interior baited with a food reward (Fig. 1. We used this MAB previously to test repeated innovation in captive hyenas; for more information about the design specifications see Johnson-Ulrich et al.^24^.

### Test protocol

We conducted all testing between 0630 to 1000 h and 1700 to 1830 h, the daylight hours during which hyenas are most active. We deployed the MAB anytime a suitable group of hyenas was located within the territories of our study clans. A suitable group was defined as one containing five or fewer hyenas within 100 m or within visible range that were either walking or resting but not engaged in hunting, border patrol, mating, courtship, or nursing behaviors. We used our research vehicle as a mobile blind to shield the researchers from the view of hyenas while we baited and deployed the MAB on the opposite side of the vehicle from hyenas. We baited the MAB with approximately 200 g of either goat or beef muscle, skin, or organ meat. During familiarization trials we also used full cream milk powder in addition to, or in place of, meat. We deployed the MAB approximately 20 m away from the nearest hyena and after MAB deployment we drove the research vehicle to a distance of 20–50 m away from the MAB. We began videotaping immediately after we deployed the MAB and we ended videotaping when we collected the MAB.

During familiarization trials we deployed the MAB with the top removed to acclimate subjects to the MAB and allow them to learn to associate the MAB with bait. Familiarization trials began when a hyena came within 5 m of the MAB and ended upon successful food retrieval (a “feed” trial) or when the hyena moved more than 5 m away from the MAB for more than 5 min. We recorded hyenas that approached the MAB, but did not make contact, as not participating in the trial. Average duration of familiarization trials was 11.7 ± 12.3 min.

If a hyena had a “feed” familiarization trial or successful test trial, and if it had moved at least 5 m away from the MAB, we immediately rebaited the MAB for successive testing. We gave hyenas successive trials as long as the testing conditions remained suitable, as described above, or until researchers ran out of bait. We did not administer successive trials following trials where every hyena that participated was unsuccessful because unsuccessful hyenas were those that had moved beyond 5 m from the MAB for more than five minutes without opening the MAB and these hyenas were extremely unlikely to spontaneously re-approach the MAB for another trial. On average, hyenas received 1.53 ± 1.25 trials per testing session and completed testing across 6.31 ± 2.58 separate sessions (Supplementary Fig. S1). Most sessions were separated by a median of 1 day (mean ± SD = 24.18 ± 60.30 days, range = 0–321 days).

We divided test trials into four different phases of testing. During Phase 1, we presented the MAB to hyenas with all four doors accessible. After a hyena had reached completion criterion for Phase 1, defined by success with the same door in three out of four consecutive trials, it progressed to Phase 2. During Phase 2, we blocked the door used in Phase 1 by bolting it shut. The same criteria for progression applied to subsequent phases until a hyena reached the criteria for progression with all four doors. We gave hyenas trials until they either reached criterion for all four doors or failed five consecutive trials during any phase of testing. We did not include hyenas that participated in fewer than five trials, of which none were successful, in our analysis. On average, hyenas participated in 9.64 ± 5.61 trials. Hyenas completed Phase 1 in 7.43 ± 2.93 trials (N = 72) either by reaching the criterion for progression or by failure, Phase 2 in 3.67 ± 1.11 trials (N = 15), Phase 3 in 4.08 ± 1.32 trials (N = 13) and Phase 4 in 4.25 ± 1.96 trials (N = 12).

We aimed to give every hyena two familiarization trials prior to being given the option to participate in test trials. On average we gave hyenas the opportunity to participate in 1.60 ± 1.54 (mean ± standard deviation) familiarization trials prior to their first Phase 1 trial, but hyenas only fed from the MAB on an average of 0.94 ± 1.11 familiarization trials prior to their first Phase 1 trial.

When we presented a group of hyenas with the MAB, we configured the MAB for the hyena at the most advanced phase of testing. For example, if one hyena in the group had progressed to Phase 3, but all the others were still on Phase 1, we would configure the MAB for the hyena on Phase 3 and block the doors that hyena had used in Phases 1 and 2. Overall, there were only five trials in total in which a hyena solved the MAB in a trial during the ‘wrong’ phase of testing by joining a trial where we configured the MAB for a group mate rather than itself. The average ‘trial group size’ per hyena per trial was 3.89 ± 3.71 hyenas (median = 3, range = 1–29). We calculated trial group size as a count of all hyenas that participated in a trial by contacting the MAB at any point in time during the trial. Overall, trial group size had a positive and significant effect on hyena participation; hyenas were slightly more likely to contact the box if there were other hyenas contacting the box (Binomial GLMM: *z* = 9.19, P < 0.001; Supplementary Table S1). We also examined the effect of ‘overall group size’ which we calculated as a count of all hyenas present within 20 m of the MAB. Overall group size had slightly negative effect on participation (Binomial GLMM: *z* = -9.81, P < 0.001; Supplementary Table S1); hyenas were slightly less likely to contact the box if there were more hyenas present within 20 m of the MAB.

Overall, we deployed the MAB 483 independent times including both familiarization trials and test trials to 280 different hyenas for a total of 2,891 observations. The dataset used in the present analysis only includes test trials from subjects that completed testing by reaching criterion for failure or subjects who had solved the MAB at least once (N = 72 hyenas, n = 694 observations). Of these 72 hyenas, 23 opened the MAB at least once (n = 318 trials). On average, we deployed the MAB 120.75 ± 25.80 times to each of our four study clans). In order to identify the 23 solvers, we deployed the MAB an average of 88.5 ± 34.72 (N = 4) times in each of our four study clans. In other words, by the 90^th^ deployment on average, we had no new subjects solve the MAB that had not already solved it at least once.

### Statistical analysis

All statistical analyses were performed using the statistical software R^48^. Here, R values were calculated for subject ID in generalized linear mixed models (GLMMs). The rptR package also allowed us to estimate uncertainty around each point estimate for R via parametric bootstrapping (n = 1,000), in which we estimated a standard error, a 95% confidence interval, and a P value for each estimate of R. P values were generated using likelihood ratio tests where model fit was compared to a null model with no grouping factor. Here, we report both adjusted R values, calculated as a ratio of the variance explained by subject ID over the residual variance, and conditional R values, calculated as the ratio of the variance explained by subject ID over the total variance, including fixed effects.

Before calculating R values for innovative problem-solving ability across doors, we created a global GLMM that included door, age class, sex, and rank as fixed effects and subject ID as a random effect in order to identify factors that might influence innovativeness. We used the glmmTMB package to create all global models^49^. To identify fixed effects of importance, we used the ‘dredge’ function in the R package MuMIn^50^. We built our final model including only the factors with large or significant effects on innovative problem-solving as fixed effects. Dredge identified nine top models for our global model on problem-solving success across doors (Δ AICc < 4). None of the included effects were estimated as important in all nine top models, but age class was included in the most top models (N = 5) and had a large effect that tended towards significance (Supplementary Table S2). Therefore, we included only age class as a fixed effect in our final model.

Likewise, before calculating R values for innovative problem-solving ability across trials, we created a global model that included age class, sex, rank, trial number, and phase of testing as fixed effects, and subject ID as a random effect. To identify fixed effects of importance we used the ‘dredge’ function in the R package MuMIn^50^. We fixed trial number and phase of testing for inclusion in all models because we wanted to control for the effects of experience and task difficulty. Dredge identified eight top models with a delta AICc of less than four (Supplementary Table S3). Here, both trial number and phase had significant effects. Once again, age class was only marginally significant but also had the largest effect size (Supplementary Table S3). Therefore we included test trial, phase of testing, and age class in all subsequent models examining problem-solving success across trials (Models 2–5.9). In Model 2, phase of testing had a significant negative effect on the likelihood of solving the MAB (GLMM: β = -0.62, SE = 0.31, *z* = -2.01, P = 0.04) which suggests that later phases of testing, where solutions that hyenas used previously were blocked, were indeed more demanding for hyenas. After controlling for the effect of phase, overall test trial number had a significant positive effect on the likelihood of solving the MAB (GLMM: β = 0.11, SE = 0.06, *z* = 1.96, P = 0.05), indicating that hyenas were more likely to solve the MAB in later than earlier trials (Fig. 6A). The positive effect of trial number could indicate that hyenas were learning how to improve their performance across trials, but this effect might also be biased by some subjects reaching the criterion to end testing (five unsuccessful trials in a row) and dropping out of the subject pool. To test this possibility we created another model where we restricted our dataset to the first ten test trials only for hyenas that had at least 10 trials, and found that test trial still had a significant positive effect on the likelihood of solving the MAB (GLMM: β = 0.30, SE = 0.10, *z* = 2.91, P = 0.004, n = 139 trials, N = 14 hyenas; Fig. 6B).

Before calculating R values all models were checked for collinearity by examining variance inflation factors (VIF). Test trial number and phase of testing consistently had VIFs > 4 in most of our models, however, we chose to include both because the high collinearity here is a result of our test protocol; hyenas only progressed to Phase 4 of testing after completing a relatively large number of trials. The main concern with high VIFs is that the estimates error and P values for the collinear factors will be increased; however, both test trial and phase of testing had consistent significant effects, which suggests that this was not a problem in our models. Next, we also examined QQ plot residuals and a histogram of the residuals using the R package DHARMa to confirm that model assumptions about the normality of residuals were not violated.

### Ethics statement

This work was conducted under research permit no. NACOSTI/P/16/35513/10422, issued by the Kenyan National Commission on Science, Technology and Innovation. The data collection procedure followed here was also approved by the Michigan State University Institutional Animal Care and Use Committee (IACUC): AUF #04/16-050-00. All research procedures were designed to adhere to the American Society of Mammalogists (ASM) Guidelines for the use of wild mammals in research and education^51^ and to the Association for the Study of Animal Behaviour (ASAB) Ethics Committee and the Animal Behaviour Society (ABS) Animal Care Committee Guidelines for the treatment of animals in behavioural research and teaching^52^.

## Supplementary information

Supplementary file 1

## Data Availability

R code and data tables used in this manuscript are available in the Knowledge Network for Biocomplexity (KNB) Data Repository (https://doi.org/10.5063/F1JQ0ZC5).

## References

[1] MacLean EL (2014). The evolution of self-control. Proc. Natl. Acad. Sci. U. S. A..

[2] Benson-Amram S, Dantzer B, Stricker G, Swanson EM, Holekamp KE (2016). Brain size predicts problem-solving ability in mammalian carnivores. Proc. Natl. Acad. Sci..

[3] Fristoe TS, Iwaniuk AN, Botero CA (2017). Big brains stabilize populations and facilitate colonization of variable habitats in birds. Nat. Ecol. Evol..

[4] Dunbar RIM, Shultz S (2007). Evolution in the social brain. Science (80-).

[5] DeCasien AR, Williams SA, Higham JP (2017). Primate brain size is predicted by diet but not sociality. Nat. Ecol. Evol..

[6] Ashton BJ, Thornton A, Ridley AR (2018). An intraspecific appraisal of the social intelligence hypothesis. Philos. Trans. R. Soc. B Biol. Sci..

[7] Cauchoix M, Hermer E, Chaine AS, Morand-Ferron J (2017). Cognition in the field: comparison of reversal learning performance in captive and wild passerines. Sci. Rep..

[8] Thornton A, Isden J, Madden JR (2014). Toward wild psychometrics: linking individual cognitive differences to fitness. Behav. Ecol..

[9] Pritchard DJ, Hurly TA, Tello-Ramos MC, Healy SD (2016). Why study cognition in the wild (and how to test it)?. J. Exp. Anal. Behav..

[10] Boogert NJ, Madden JR, Morand-Ferron J, Thornton A (2018). Measuring and understanding individual differences in cognition. Philos. Trans. R. Soc. B Biol. Sci..

[11] Cauchoix M (2018). The repeatability of cognitive performance: a meta-analysis. Philos. Trans. R. Soc. B Biol. Sci..

[12] Bell AM, Hankison SJ, Laskowski KL (2009). The repeatability of behaviour: a meta-analysis. Anim. Behav..

[13] Morand-Ferron J, Cole EF, Quinn JL (2015). Studying the evolutionary ecology of cognition in the wild: a review of practical and conceptual challenges.

[14] van Horik JO, Madden JR (2016). A problem with problem solving: motivational traits, but not cognition, predict success on novel operant foraging tasks. Anim. Behav..

[15] Cauchoix M, Chaine AS, Barragan-Jason G (2020). Cognition in context: plasticity in cognitive performance in response to ongoing environmental variables. Front. Ecol. Evol..

[16] Reader, S. & Laland, K. *Animal innovation*. (Oxford University Press, 2003).

[17] Shaw RC (2017). Testing cognition in the wild: factors affecting performance and individual consistency in two measures of avian cognition. Behav. Processes.

[18] Cole EF, Cram DL, Quinn JL (2011). Individual variation in spontaneous problem-solving performance among wild great tits. Anim. Behav..

[19] McCune KB, Jablonski P, Lee S, Ha RR (2019). Captive jays exhibit reduced problem-solving performance compared to wild conspecifics. R. Soc. Open Sci..

[20] Ashton BJ, Ridley AR, Edwards EK, Thornton A (2018). Cognitive performance is linked to group size and affects fitness in australian magpies. Nature.

[21] Holekamp KE, Sakai ST, Lundrigan BL (2007). The spotted hyena (*Crocuta crocuta*) as a model system for study of the evolution of intelligence. J. Mammal..

[22] Benson-Amram S, Holekamp KE (2012). Innovative problem solving by wild spotted hyenas. Proc. R. Soc. B Biol. Sci..

[23] Benson-Amram S, Weldele ML, Holekamp KE (2013). A comparison of innovative problem-solving abilities between wild and captive spotted hyaenas *Crocuta crocuta*. Anim. Behav..

[24] Johnson-Ulrich L, Johnson-Ulrich Z, Holekamp K (2018). Proactive behavior, but not inhibitory control, predicts repeated innovation by spotted hyenas tested with a multi-access box. Anim. Cogn..

[25] Johnson-Ulrich L, Benson-Amram S, Holekamp KE (2019). Fitness consequences of innovation in spotted hyenas. Front. Ecol. Evol..

[26] Nakagawa S, Schielzeth H (2010). Repeatability for gaussian and non-gaussian data: a practical guide for biologists. Biol. Rev..

[27] Griffin AS, Guillette LM, Healy SD (2015). Cognition and personality: an analysis of an emerging field. Trends Ecol. Evol..

[28] Stoffel MA, Nakagawa S, Schielzeth H (2017). rptR: repeatability estimation and variance decomposition by generalized linear mixed-effects models. Methods Ecol. Evol..

[29] Huebner F, Fichtel C, Kappeler PM (2018). Linking cognition with fitness in a wild primate: fitness correlates of problem-solving performance and spatial learning ability. Philos. Trans. R. Soc. B Biol. Sci..

[30] Thornton A, Samson J (2012). Innovative problem solving in wild meerkats. Anim. Behav..

[31] Manrique HM, Völter CJ, Call J (2013). Repeated innovation in great apes. Anim. Behav..

[32] Huebner F, Fichtel C (2015). Innovation and behavioral flexibility in wild redfronted lemurs (*Eulemur rufifrons*). Anim. Cogn..

[33] Borrego N, Dowling B (2016). Lions (*Panthera leo*) solve, learn, and remember a novel resource acquisition problem. Anim. Cogn..

[34] Chow PKY, Lea SEG, Leaver LA (2016). How practice makes perfect: the role of persistence, flexibility and learning in problem-solving efficiency. Anim. Behav..

[35] Lermite F, Peneaux C, Griffin AS (2017). Personality and problem-solving in common mynas (*Acridotheres tristis*). Behav. Process..

[36] Ducatez S, Audet JN, Lefebvre L (2015). Problem-solving and learning in carib grackles: individuals show a consistent speed–accuracy trade-off. Anim. Cogn..

[37] Griffin AS, Guez D (2014). Innovation and problem solving: a review of common mechanisms. Behav. Processes.

[38] Diquelou MC, Griffin AS, Sol D (2015). The role of motor diversity in foraging innovations: a cross-species comparison in urban birds. Behav. Ecol..

[39] Chow PKY, Lea SEG, Hempel de Ibarra N, Robert T (2017). How to stay perfect: the role of memory and behavioural traits in an experienced problem and a similar problem. Anim. Cogn..

[40] Smith JE, Holekamp KE (2018). Spotted hyenas. *Encycl*. Anim. Behav..

[41] Rowe C, Healy SD (2014). Measuring variation in cognition. Behav. Ecol..

[42] Reader SM, Morand-Ferron J, Flynn E (2016). Animal and human innovation: novel problems and novel solutions. Philos. Trans. R. Soc. B Biol. Sci..

[43] Van Horn RC, McElhinny TL, Holekamp KE (2003). Age estimation and dispersal in the spotted hyena (*Crocuta crocuta*). J. Mammal..

[44] Engh AL, Esch K, Smale L, Holekamp K (2000). Mechanisms of maternal rank ‘inheritance’ in the spotted hyaena *Crocuta crocuta*. Anim. Behav..

[45] Holekamp, K. E. & Dloniak, S. M. Intraspecific variation in the behavioral ecology of a tropical carnivore, the spotted hyena. in *Behavioral ecology of tropical animals* vol. 42 189–229 (Elsevier, 2010).

[46] Holekamp KE, Smale L, Szykman M (1996). Rank and reproduction in the female spotted hyaena. Reproduction.

[47] Auersperg AMI, Gajdon GK, von Bayern AMP (2012). A new approach to comparing problem solving, flexibility and innovation. Commun. Integr. Biol..

[48] R Core Team. R: A language and environment for statistical computing. (2019).

[49] Brooks ME (2017). glmmTMB balances speed and flexibility among packages for zero-inflated generalized linear mixed modeling. Res. J..

[50] Bartoń, K. MuMIn: Multi-model inference. (2018).

[51] Sikes RS (2016). 2016 guidelines of the american society of mammalogists for the use of wild mammals in research and education. J. Mammal..

[52] Behaviour, A. Guidelines for the treatment of animals in behavioural research and teaching. *Anim. Behav.* **123**, I–IX (2017). 10.1006/anbe.1999.1349 10640387

