# Supplementary material for: Innovative problem-solving in wild hyenas is reliable across time and contexts
Source: Sci Rep. 2020 Aug 3;10:13000. doi: 10.1038/s41598-020-69953-5 (PMC7400572; doi:10.1038/s41598-020-69953-5)
Supplement: Supplementary file 1 [file 41598_2020_69953_MOESM1_ESM.pdf]

Innovative problem-solving in wild hyenas is reliable across time and contexts

1. Lily Johnson-Ulrich[1,2*]

2. Kay E Holekamp[1,2]

3. David Z Hambrick[3]

[1]Department of Integrative Biology, Michigan State University, East Lansing, MI, 48824, USA
[2]Ecology, Evolutionary Biology, & Behavior Program, Michigan State University, East Lansing, MI, 48824, USA
[3]Department of Psychology, Michigan State University, East Lansing, MI, 48824, USA
[*]john3923@msu.edu
Corresponding author:
Lily Johnson-Ulrich
288 Farm Lane, Rm 203
Natural Sciences Bldg
East Lansing, MI 48823
U.S.A.
+1 301 676 4128
john3923@msu.edu

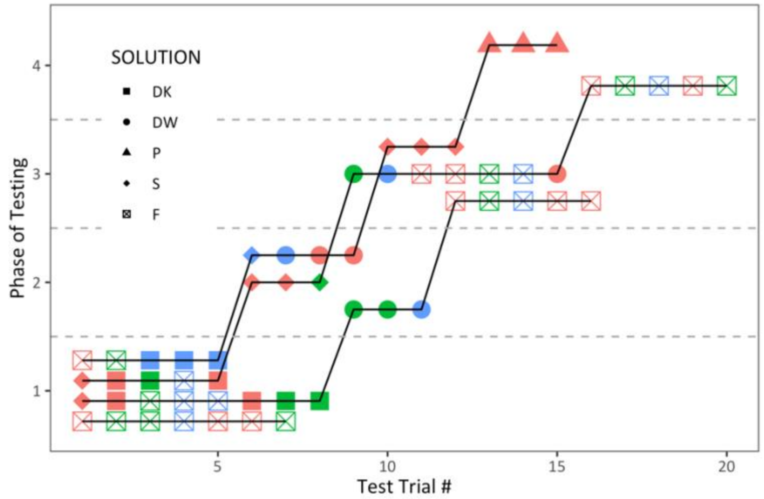

Supplementary Figure S1. Visualization of test trial results for four hyenas across the four phases of testing. Each line represents a single hyena. Shapes indicate the door used to open the MAB (DK = door knob door; DW = drawer, S = sliding door, P = push door) or failure (F) to open any of the four doors on each trial. Adjacent shapes with the same color indicate trials that took place within the same testing session whereas adjacent shapes with different colors indicate trials that were separated in different testing sessions. Test trial # indicates the temporal order in which trials occurred; trials within the same session occurred immediately following previous trials, but the amount of time between trials in different sessions varied.

Factor	β	SE	*z*	P
Park – Triangle/Reserve	0.23	0.24	0.94	0.35
Sex	-0.32	0.24	-1.33	0.18
Age class – cub	1.28	0.27	4.74	**<0.001**
Age class – subadult	1.52	0.27	5.59	**<0.001**
Rank	-0.29	0.12	-2.54	**0.011**
# prior feeds	0.54	0.08	7.12	**<0.001**
# prior solves	0.15	0.04	3.89	**<0.001**
Trial #	-0.05	0.01	-5.41	**<0.001**
Familiarization vs test trial	0.13	0.12	1.12	0.27
Body condition – fat	0.14	0.21	0.67	0.50
Body condition – gaunt	-0.21	0.62	-0.33	0.74
Body condition – bloody	-0.68	1.12	-0.61	0.54
Bait – milk powder	0.08	0.19	0.43	0.67
Bait – bone	0.05	0.15	0.31	0.76
Bait – muscle tissue	0.03	0.19	0.17	0.86
Bait – offal	0.06	0.21	0.27	0.79
Bait – spoiled	0.18	0.18	1.02	0.31
Location – carcass	0.55	0.37	1.48	0.14
Location – den	0.10	0.17	0.56	0.58
# hyenas present within 20m of the MAB	-0.17	0.02	-9.81	**<0.001**
# hyenas present contacting the MAB	0.24	0.03	9.19	**<0.001**
Migration present/absent	-0.06	0.16	-0.36	0.72

Supplementary Table S1. Factors affecting participation in trials with the MAB. The data used for this model includes the complete dataset from which innovation and reliability data were extracted. This dataset included trials from both test and familiarization trials (425 trials conducted with 263 hyenas for a total of 2429 observations of participation in trials). Participation was coded as a binary variable with a 1 indicating that a hyena made contact with the MAB with any body part and a 0 indicating that the hyena approached the MAB within 20m but did not make contact. Significant P values are bolded.

Factor	β	P	N
Age Class – Cub	-3.02	0.06	5
Age Class – Subadult	-2.65	0.06	5
Rank	0.71	0.28	4
Sex – Male	0.42	0.72	4
Solution – Drawer	-1.09	0.22	1
Solution – Push	-1.42	0.12	1
Solution – Slide	-0.75	0.40	1

Supplementary Table S2. Results from model averaging the top nine models (Δ AICc < 4) identified by the R package MuMIn for innovative problem-solving across doors. Estimates and p-values are estimated by averaging only models in which factors were included (conditional average).

Factor	β	P	N
Age Class – Cub	-1.15	0.10	4
Age Class – Subadult	-1.07	0.06	4
Rank	0.46	0.11	4
Sex – Male	-0.12	0.85	4
Test Trial #	0.12	0.03	8
Phase #	-0.69	0.03	8

Supplementary Table S3. Results from model averaging the top eight models (Δ AICc < 4) identified by the R package MuMIn for innovative problem-solving across trials. Estimates and p-values are estimated by averaging only models in which factors were included (conditional average).
